# Effect of Plant Protein Ingredients at a Range of Pre-Hydration Levels on Technological Properties of Hybrid Beef Patties

**DOI:** 10.3390/foods14172957

**Published:** 2025-08-25

**Authors:** Zuo Song, Joseph P. Kerry, Rahel Suchintita Das, Brijesh K. Tiwari, Antonia Santos, Ruth M. Hamill

**Affiliations:** 1 Teagasc Food Research Centre, Ashtown, D15 DY05 Dublin, Ireland; zuo.song@teagasc.ie (Z.S.); rahel.suchintitadas@teagasc.ie (R.S.D.); brijesh.tiwari@teagasc.ie (B.K.T.); antonia.santos@teagasc.ie (A.S.); 2 Food Packaging and Material Sciences Group, School of Food and Nutritional Sciences, University College Cork, T12 E138 Cork, Ireland; joe.kerry@ucc.ie

**Keywords:** alternative plant protein, hybrid formulation, texture, water-holding capacity

## Abstract

Hybrid plant and meat (HPM) products, in which a portion of meat is substituted with alternative plant protein-containing ingredients, offer a promising option for flexitarian consumers seeking to increase plant protein consumption while continuing to enjoy the sensory qualities of meat products. This study evaluated the effects of faba bean protein (FBP), pea protein (PP), and rice protein (RP) ingredients at a 12.5% meat protein substitution level, under varying pre-hydration conditions and, subsequently, on the technological properties of hybrid plant/beef patties (HPBP). Colour measurements indicated that plant protein ingredient addition to HPBP resulted in increased lightness (L*) and decreased redness (a*) values. HPBP showed reduced cooking loss compared to 100% beef patties, and cooking loss increased with higher pre-hydration levels of plant proteins. Faba bean hybrid patty (FBHP) exhibited lower texture scores, while the patty containing non-hydrated RP had the highest hardness values. The texture of patties with PP was comparable to the control, irrespective of the hydration status of the plant protein. Inclusion of plant proteins also reduced water mobility by restricting intracellular water. Overall, these findings provide valuable insights into the selection of suitable plant proteins and the requirement for optimal pre-hydration of plant proteins prior to incorporation into HPBP to ensure optimal technological properties.

## 1. Introduction

In recent years, the global food sector has experienced significant growth in the demand for meat analogues and meat-reduced products [1]. This growth is closely linked to concerns about the carbon footprint of livestock production [2,3,4,5], coupled with reported nutritional concerns relating to overconsumption of processed meats [6]. However, t the inferior textural properties and flavours of plant-based meat analogues [7], along with consumer unfamiliarity and scepticism regarding the sensory aspects of meat substitutes [8], hinder consumer acceptance of meat analogues [9]. Therefore, transitioning from a meat-based diet to a strict vegan or vegetarian diet may be challenging [3]. Hybrid plant and meat (HPM) products, which replace a substantial portion of animal protein with alternative protein ingredients, help bridge the acceptability gap for flexitarians. These products facilitate meat eaters to adopt a meat-reduced diet by increasing their familiarity with meat substitutes, which may support the transition to more plant-based diets [10]. The HPM approach has the potential to closely replicate the sensory experience of traditional meat products [1,7,11]. Additionally, HPM products combine the potential nutritional benefits of both meat-based and plant-based ingredients [11]. This approach addresses the concerns outlined above but also allows for meat to continue to play an important and pivotal role, providing high nutritional density options, balance, and food choices for consumers.

In general, a positive correlation has been reported to exist between fibre content in HPM products and the proportion of plant-based ingredients incorporated [12,13,14]. Additionally, multiple studies have demonstrated that hybrid sausages [15,16,17], meatballs [18], and hybrid patties [11,19,20,21,22,23] have significantly reduced cooking loss compared to 100% meat products. This reduction may be attributed to the strong water-binding capability of plant proteins and the presence of other plant-derived hydrocolloids, effectively enhancing water and fat retention. However, the majority of studies [12,15,24] have shown that incorporating various meat substitutes and levels of substitution applied in HPM products tends to diminish the desirable texture properties associated with meat products. Broucke, Van Poucke [24] formulated hybrid pork sausages in which 20% of pork meat was substituted with pea protein isolate, low-moisture pea extrudate, and high-moisture pea extrudate, showing that the inclusion of pea proteins in the hybrid meat matrix weakened network formation despite providing a complete amino acid profile. Nie, Xiong, and Jiang [25] investigated the effects of formulation on the textural and microstructural properties of hybrid pork sausage incorporating functionalized pea protein isolate and pea flour at 25% and 50% levels, reporting that hardness and breaking force declined with increasing substitution levels. Textural properties are, therefore, frequently considered the most challenging characteristic limiting the effectiveness in replicating the attributes of meat products with plant-based alternatives. The effects of adding plant-based ingredients on the texture properties of HPM products are complex and influenced by factors such as water content, nutrient composition, the inclusion ratio and format of the plant-based ingredients used, meat sources utilised, and the methods employed during processing [16]. Therefore, the careful selection of plant-based ingredients as meat replacers is crucial. This selection must also consider well-established nutritional benefits and techno-functional properties.

Faba bean (*Vicia faba* L.) is valued not only for its well-balanced amino acid profile and nitrogen-fixing properties but also for its potential in food applications due to faba bean protein exhibiting similar physical and functional properties to soybean protein [26,27]. Its major proteins are globulins, which are soluble in dilute salt solutions, and primarily consist of two high-molecular-weight proteins known as legumin and vicilin (11S and 7S) [28]. The legumin-to-vicilin ratio plays a key role in determining functional properties of proteins [26]. Pea (*Pisum sativum*) is a rich source of protein, starch, and fibre, and its protein is widely used due to its low allergenicity, mild taste, and high protein content [27]. Globulin and albumin are the two major protein types in pea protein, and their composition, molecular structure, and charge distribution determine the physical and chemical properties of PP. Physical and chemical properties of PP can significantly influence its behaviours in food processing [29]. It has also been shown to exhibit excellent functional attributes, including water and oil holding capacity, emulsification, and viscosity enhancement [30]. Rice (*Oryza sativa*) contains a significant amount of protein, consisting of four different fractions, namely, prolamin, glutelin, globulin, and albumin, with varying solubility characteristics. Glutelin, the major fraction, can impact WHC due to its solubility characteristics [31]. Rice protein (RP) presents significant potential as a nutritious, affordable, and widely available protein source. It is recognised as a valuable food ingredient due to its balanced amino acid composition, high biological value, hypoallergenic nature, and diverse functional properties [31].

In addition to the intrinsic functional and nutritional properties of plant protein ingredients, the interactions between water and protein are among the most important determinants impacting the functional role of proteins in foods [32]. For example, the hydration level of plant proteins prior to application in hybrid products has also been shown to be influential on quality performance when developing meat analogues [33]. Current studies have shown that plant proteins possess favourable water-binding properties [34]. However, it must be noted that these studies focused on the binding of water to the extracted plant proteins, while in gelled food products, such as HPM products, there is not yet enough information about the effect of pre-hydration of plant proteins on the properties of HPM products. For this reason, the present study sought to investigate the impact of plant protein pre-hydration level on the technological properties of hybrid beef patties.

The objective of this study is to assess the technological properties of hybrid beef patties formulated with three different plant protein ingredients (FBP, PP, and RP) at a 12.5% meat protein substitution level, and to investigate the effect of varying pre-hydration levels of these proteins on key functional parameters. Differences in cooking loss, water-holding capacity, colour, water mobility, and texture profile were measured. Specifically, consumer surveys have shown that meat alternatives, which closely resemble the texture of animal-based meat products, are more likely to be accepted [35,36]. Therefore, this study aimed to identify which hybrid formulations produced texture profiles most comparable to the 100% beef control patties. This study presents a novel approach to evaluating the applicability of plant-based ingredients in hybrid meat products and aims to provide actionable insights for processors targeting the development and optimisation of HPM products in the food industry.

## 2. Materials and Methods

### 2.1. Materials

Beef (95% visual lean) and beef fat were purchased from Kepak (Dublin, Ireland). Having trimmed the connective tissue and visible fat, the beef was crudely diced and stored, along with the fat, at −20 °C for further use. The protein, fat, moisture, and ash contents of the trimmed beef were estimated by Perten (DA 7250TM NIR, PerkinElmer, Norwalk, CT, USA) as 21.1 ± 0.5 g/100 g, 5.7 ± 1.0 g/100 g, 71.7 ± 1.8 g/100 g, and 1.9 ± 0.1 g/100 g, respectively, and these values were used for the subsequent formulation design. Faba bean (*Vicia faba* L., variety ‘Lynx’) protein (FBP) was extracted with the alkali extraction and acid precipitation method [37]. Pea protein (PP) (ProDiem Pea 7028, lot 0005509976) and rice protein (RP) (ProDiem Rice 5020, lot 0006070056) were provided by Kerry Group (Naas, Ireland).

### 2.2. Characteristics of Plant Protein Ingredients

Moisture content of plant protein powder was determined using TGM800 Thermogravimetric Moisture Determinator (LECO, St. Joseph, MI, USA) according to the AOAC official method 925.10 [38]. Fat content was analysed using the NMR Smart Trac rapid analyser (CEM Corporation, Matthews, NC, USA) according to the AOAC method 2008.06 [39]. Protein was determined using a LECO FP628 (LECO Corporation, St. Joseph, MI, USA) according to the AOAC method 992.15 [40]. Ash was determined using an muffle furnace (Carbolite, Sheffield, UK) at 550 °C according to AOAC 920.153 [41]. The percentage of carbohydrates was determined by subtracting the total percentage of moisture, fat, protein, and ash from 100. Water activity (a_w_) was determined using the Water Activity Meter model AquaLab Pre (Labcell, Medstead, UK) according to AOAC 978.18 [42]. All pH measurements of plant proteins were taken using a slurry made by adding 3 g of the ingredient protein to 27 mL of distilled water using a pH meter (Hanna Instruments Ltd., Leighton Buzzard, UK). Each analysis was conducted in triplicate using three separate ingredient samples.

Water- and oil-binding capacities of plant protein powders were determined based on the method described by Malik and Saini [43]. A total of 1 g of protein isolate was mixed with 10 mL of distilled water or sunflower oil and stirred for 5 min. The mixture was then allowed to stand for 30 min before being centrifuged for 30 min at 4143× *g* using a Sorvall LYNX 6000 centrifuge (Thermo Fisher Scientific, Dublin, Ireland). Following centrifugation, the supernatant was discarded, and the tubes were inverted at a 45° angle for 25 min to drain any remaining liquid from the protein sediment. The water- and oil-binding capacities were calculated as follows:

(1) Water-binding capacity (WBC) or Oil-binding capacity (OBC) = (The mass of the tube with plant protein powder and absorbed water/oil – The mass of the tube and plant protein powder)/The mass of the plant protein powder

### 2.3. Preparation and Forming of Beef and Hybrid Meat Patties

**Figure 1:**
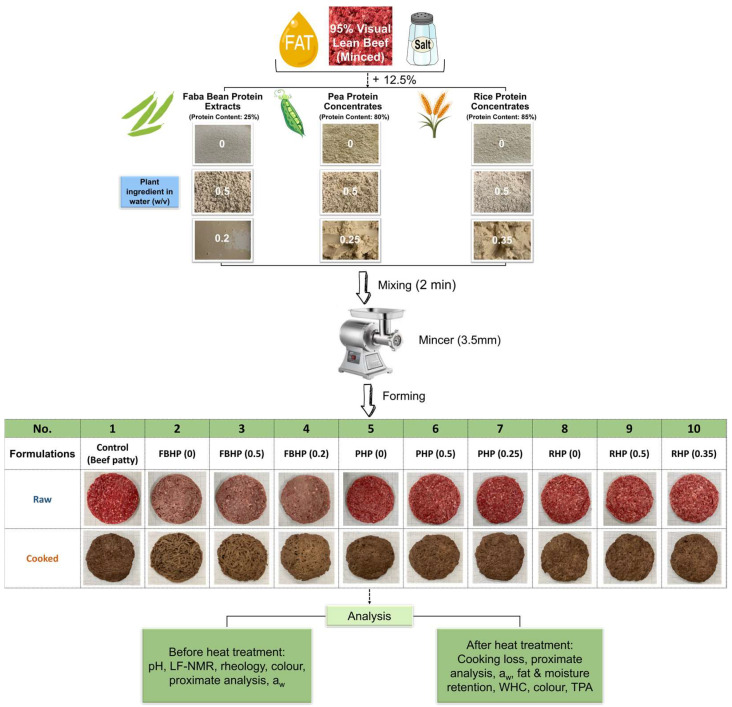
Workflow of the experiment. FBHP, PHP, and RHP represent faba bean, pea, and rice hybrid patties, respectively.

One control and nine experimental beef patty formulations were prepared following the procedure outlined in Figure 1. Based on formulation design, 12.5% of meat protein in beef patties was substituted with dry or hydrated FBP (plant ingredient in water (*w*/*v*): 0.5, 0.2), PP (plant ingredient in water (*w*/*v*): 0.5, 0.25), and RP (plant ingredient in water (*w*/*v*): 0.5, 0.35). Before processing, frozen beef and fat from the same batch were thawed at 4 °C overnight. Thawed lean beef and fat were separately minced through a mincer (MAINCA, Barcelona, Spain) employing a 9 mm mincing plate. The coarse-minced lean beef was mixed for 3 min using a mixer (GJ464–BUFFALO Planetary Mixer 7L, Bristol, UK) at speed level 1.

To ensure a consistent fat content of 10% across all formulations, the control sample (Table 1) was prepared by mixing 94% coarse-minced lean beef, 5% added fat, and 1% salt for 2 min at speed level 1. For the faba bean hybrid patty (FBHP), pea hybrid patty (PHP), and rice hybrid patty (RHP), the plant protein was hydrated with water and left to hydrate overnight. Ingredients (1% salt and an appropriate amount of fat and plant protein slurry) were added to the mixed coarse-minced lean beef and mixed for 2 min at speed level 1. Once the mixture was well blended and homogenous in appearance, each formulation was minced through a 3.5 mm mincing plate. Around 100 g of each mixture was transferred to a burger former to create round-shaped burger patties of diameter 10 cm and height 1 cm. Samples were then cooled in the blast-freezer (−18 °C) for one hour, vacuum-packed, and stored at −18 °C until required for further analysis.

### 2.4. Measurement of pH in Beef and Hybrid Patties

A total of 5 g of raw patty material was homogenised (T 25 digital ULTRA-TURRAX^®^, IKA, Staufen, Germany) for 2 min with 45 mL of distilled water. A portable pH meter (Hanna Instruments Ltd., Leighton Buzzard, UK), equipped with built-in temperature compensation, was calibrated using pH buffers (4.0, 7.0, and 10.0) before the measurement [44]. The pH of the homogenate was determined by inserting the probe of the pH meter directly into the sample. Readings were taken in triplicate.

### 2.5. Low-Field Nuclear Magnetic Resonance (LF-NMR)

LF-NMR was used to assess the mobility and distribution of water in the samples by measuring the spin–spin relaxation time (T_2_) of hydrogen protons [39]. Measurements were performed on raw samples using a Maran Ultra NMR spectrometer (Oxford Instruments Molecular Biotools Ltd., Abington, UK), following the method of McDonnell et al. [45]. A total of 10 g of raw sample per formulation was placed in cylindrical glass tubes (1–1.4 cm in diameter) in a block heater at 25 °C for 1 h prior to analysis. Every measurement taken was the result of 16 scan repetitions. The T_2_ relaxation data were obtained through multi-exponential fitting of the T_2_ relaxation data using the RI Win-DXP programme (Oxford Instruments Molecular Biotools Ltd., Abington, Oxfordshire, UK). The resulting T_2_ components were identified as T_2b_ (bound water), T_21_ (immobilised water), and T_22_ (free water), based on their relaxation times. The relative proportion of each water population (P_2b_, P_21_, and P_22_) was calculated from the area under the corresponding curve peaks.

### 2.6. Rheology

Rheological measurements were conducted using a controlled stress rheometer (MCR 301, Anton Paar Gmbh, Graz, Austria) equipped with a 25 mm serrated parallel probe/base plate. The raw sample was loaded onto the rheometer base. Excess sample was trimmed, and the edges were covered with petroleum jelly. The test was performed with a frequency ranging from 0.01 to 10 Hz, a constant shear strain of 0.01%, a 2 mm gap, and a 5 min waiting time. The plate was covered with a Peltier hood to maintain a temperature of 25 °C. The linear viscoelastic region was assessed preliminarily with an amplitude sweep (ranging from 0.001 to 0.05%). The storage modulus (G′) and loss modulus (G′′) of the samples were recorded.

### 2.7. Cooking Loss

Beef or hybrid patties were cooked in a preheated oven at 160 °C for 6 min, flipped and cooked until the burgers reached an internal core temperature of 72 ± 2 °C [46]. The temperature of burgers was evaluated using a digital thermometer (SensorTech Ltd., Dublin, Ireland). Visible exudates were removed gently using a paper towel after cooking. Cooking loss from the patty was measured by weighing the initial individual weight of the raw patty and, subsequently, the final weight of the same cooked patty, according to the following formula [47,48].

(2) Cooking loss (%) = (Initial weight – Final weight)/(Initial weight) × 100

### 2.8. Proximate Analyses and Water Activity of Raw and Cooked Patties

Proximate analyses were conducted on samples defrosted overnight at 4 °C. Samples were homogenised using a grinder (Cookworks, Argos Limited, Milton Keynes, UK) before analysis. The moisture content of the cooked sample was evaluated by weighing it before and after drying in an oven (Gallenkamp Labs, Cambridge, UK) at 105 °C overnight. Fat, protein, ash content, moisture of raw samples, and a_w_ were measured according to methods described in Section 2.2.

### 2.9. Moisture Retention, Fat Retention, and Water-Holding Capacity (WHC) of Patties

The moisture retention value represents the amount of moisture retained in the cooked patty per 100 g of sample. The moisture retention in the beef/hybrid patty after cooking was determined according to the following equation [49]:

(3) Moisture retention (%) = (% Yield × % moisture in cooked patty)/100

The fat retention value represents the amount of fat retained in the patty after cooking, and it was calculated using the following equation [50]:

(4) Fat retention (%) = (Cooked patty weight × (% fat in cooked patty))/(Raw patty weight × (% fat in raw patty)) × 100

The WHC of all patties were measured using the method described by Thangavelu et al. [51] with some modifications. Raw samples (approximately 10 g, weight A) were weighed in a 50 mL centrifuge tube and heated in a water bath at 90 °C for 10 min. Samples were wrapped with cheesecloth after cooling to room temperature. Samples were placed in a centrifuge tube with 1/3 cotton wool at the bottom of each tube and centrifuged for 10 min at 9000× *g* for 10 min at 4 °C using a Sorvall Lynx 6000 centrifuge (Fischer Scientific Ireland, Dublin, Ireland). The centrifuged samples were reweighed (weight B), and the WHC was calculated using the following equations:

(5) Water-holding capacity (WHC) % = 1 – (Weight A-Weight B)/(Weight A × % Moisture of the sample) × 100

### 2.10. Colour Measurements

Instrumental colour of raw and cooked patty samples was measured using an UltraScan Pro (Hunter Associates Laboratory, Inc., Reston, VA, USA) dual beam xenon flash spectrophotometer, with a viewing port of 25 mm and a standard illuminant D65 with an observer angle of ten degrees. The calibration was carried out using a light trap (L = 0) and a standard white tile (L = 100; X = 88.69; Y = 93.58; Z = 100.45), covered with transparent cling film. The samples were wrapped in cling film (Bakewell Premium Cling Film) to bloom for 30 min prior to measurement. The colour results were expressed as L* (lightness/darkness), a* (redness/greenness), and b* (yellowness/blueness). The average of nine readings per treatment was recorded.

The redness index (RI), calculated as a*/b*, was used to evaluate the red hue of raw patties, as described by Chen, Chiu and Huang [52]. To determine the degree of surface browning in cooked burgers, the browning index (BI) was calculated using the following equations:

(6) BI = 100 × ((x – 0.31)/0.17)

where

(7) x = (a* + 1.75 × L*)/(5.645 × L* + a* − 3.012 × b*)

### 2.11. Texture Profile Analysis (TPA)

Texture Profile Analysis was performed on cooked beef patties based on a method described by Bourne [53]. The values of hardness (N), cohesiveness, springiness (mm), and chewiness (N·mm) were obtained using an Instron universal testing machine (model 5543, Instron (UK) Ltd., High Wycombe, UK). Cylindrical samples were taken from random locations in cooked patties using a No. 18 cork-borer (26.25 mm), and each sample was compressed twice axially to 90% of its original height in a two-cycle compression test. The TPA was performed using a 500 N load cell and a crosshead speed of 100 mm/min. Hardness was defined as the peak force during the first compression cycle, while cohesiveness was the ratio of the positive area under the force–time curve of the second compression to that of the first. Springiness represented the distance the sample recovered between the end of the first and the start of the second compression. Chewiness was defined as hardness × cohesiveness × springiness.

### 2.12. Statistical Analysis

Two independent batches of each patty formulation were prepared. Within each batch, all beef/hybrid patty tests were performed at least in triplicate on each occasion (nine replicates in the case of colour analysis), and mean values were reported. Treatments were compared by one-way analysis of variance (ANOVA) followed by Duncan’s test (*p* < 0.05) using SPSS (IBM SPSS Statistics 29.0). For data exploration of texture variables, Principal Component Analysis (PCA) for texture-related parameters was applied, following mean centering and scaling to unit variance.

## 3. Results and Discussion

### 3.1. Composition and Techno-Functional Attributes of Plant Proteins

Significant differences (*p* < 0.05) in chemical composition, a_w_, pH, WBC, and OBC were observed between FBP, PP, and RP ingredients (Table 2). RP had a significantly (*p* < 0.05) higher fat content than FBP and PP, while PP exhibited the lowest moisture content and a_w_. Protein contents ranged from 24.70 to 81.14 g/100 g, with FBP showing the lowest value and RP the highest. FBP had the highest carbohydrate content (60.53 g/100 g), followed by RP (12.51 g/100 g) and PP (2.79 g/100 g). Ash content ranged from 1.09 to 5.54 g/100 g, with FBP containing the highest amount. Additionally, FBP had a lower pH (4.90) compared to PP (7.52) and RP (6.74). The low pH of FBP is likely due to its isolation via isoelectric precipitation, which reduces the solution’s pH to 4.5 [37]. WBC and OBC refer to the amount of water and oil, respectively, that can be retained per unit weight, indicating ingredient capacity to prevent fluid loss during storage or processing [54]. High WBC is associated with softer textures. In this study, WBC ranged from 1.73 to 4.70 g/100 g (dry basis), with PP showing the highest WBC and RP the lowest. The high WBC of PP aligns with findings by Shoaib, Sahar [55], who reported that PP can absorb up to three times its weight in water. In contrast, the low WBC of RP likely results from its poorer water solubility [27], attributed to its major protein component, glutelin (commonly referred to as glutenin), which constitutes about 80% of RP content [56]. This hydrophobic protein maintains its structure through disulfide bonds and hydrophobic interactions, which contribute to its insolubility [56]. OBC, which is important for enhancing mouthfeel and retaining flavour in food formulations [57], was lowest in PP and highest in FBP. Overall, the compositional and techno-functional variations observed among FBP, PP, and RP can be attributed to factors such as cultivar type, extraction and drying methods, and whether the proteins were produced in a laboratory or obtained as commercial ingredients [58,59]. The techno-functional behaviour of these proteins highlights their potential in hybrid meat products, although soy protein remains a widely used benchmark in plant-based meat formulations [60].

### 3.2. pH

Assessing pH is crucial for evaluating the eating quality of meat, as it significantly influences WHC, which, in turn, affects the microstructure and texture of meat products [12]. Ultimate pH of beef typically ranges from pH 5.4 to 5.8 [61]. In this study, substituting beef with PP and RP did not result in significant (*p* > 0.05) changes in pH values compared to the control (Table 3). This is likely due to the inherently neutral pH of both PP and RP [12] and aligns with previous findings where the pH of RP-enriched hybrid meat emulsions remained similar to that of the control [1]. In contrast, patties formulated with FBP showed significantly lower pH values compared to the control (*p* < 0.05), ranging from 5.36 to 5.55. These findings reinforce the idea that the pH of HPM products is influenced by the intrinsic pH of the incorporated plant-based ingredients [12,20,62]. An increase in pH can enhance the emulsion stability of HPM products by improving the WBC of plant proteins and moving away from the isoelectric points of both meat and plant proteins [1]. Additionally, raising the pH of the meat batters to around six or higher has been suggested as a strategy to overcome product softness [61]. However, this approach could raise food safety concerns, as a more neutral pH value is associated with greater susceptibility to food spoilage and the presence of food pathogens [63].

### 3.3. Water Mobility as Measured Using LF-NMR

**Figure 2:**
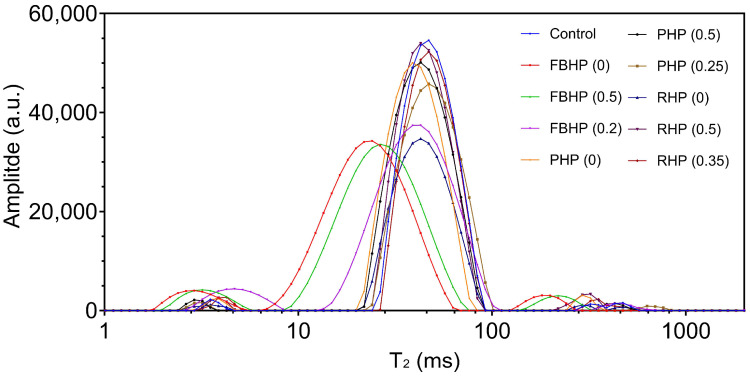
Distribution of the transverse relaxation time (T_2_) spectra of beef/hybrid patties. FBHP, PHP, and RHP represent faba bean, pea, and rice hybrid patties, respectively. Each data point represents an average of runs.

Three peaks were identified in the patties (Figure 2), and the population distribution under each curve (P_2b_, P_21_, and P_22_) is presented in Table 4. The control beef patty showed a dominant T_21_ peak (47.12 ms), indicating that immobilised water was the main fraction. Smaller T_2b_ and T_22_ peaks (3.64 ms and 420.74 ms, respectively) represented the bound and free water fractions were also observed. In contrast, the hybrid patty with non-hydrated FBP (FBHP (0)) exhibited a leftward shift in the T_21_ peak, indicating reduced water mobility. This likely reflects stronger water binding within the hybrid matrix due to the presence of water-binding components (e.g., fibre, starch) in FBP, which restricts water movement [58]. This observation is consistent with findings by Lin and Barbut [64], who reported that plant proteins can effectively trap water within the hybrid meat matrix, resulting in a lower proportion of free water compared to lean meat batter. However, as the pre-hydration level of FBP increased (to 0.2 and 0.5 *w*/*v*), the T_21_ peak shifted toward longer T_2_ times, indicating that water became more mobile and less tightly bound. This trend corresponded with decreased WHC and increased cooking loss, suggesting weaker interactions between water and protein matrix at higher hydration levels. Similar trends were observed in patties formulated with PP and RP. Increased pre-hydration of these plant proteins led to higher T_21_ and T_22_ values, indicating a shift toward more mobile water fractions. This change in water dynamics further supports the hypothesis that excessive hydration of plant proteins can weaken the overall water retention within the meat matrix.

### 3.4. Rheological Properties

**Figure 3:**
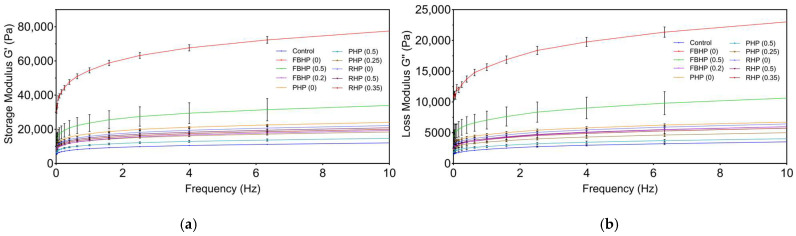
(**a**) Storage modulus (G′) and (**b**) loss modulus (G″) of beef/hybrid patties. FBHP, PHP, and RHP represent faba bean, pea, and rice hybrid patties, respectively. Data are presented as mean ± standard deviation.

To investigate textural characteristics of plant-based hybrid patties in a more detailed manner, rheological measurements were conducted on beef and hybrid meat batters at a constant temperature of 25 °C (Figure 3). The storage modulus (G′), which reflects the elastic or solid-like properties of the sample, is associated with mechanical strength and shape maintenance [65], whereas the loss modulus (G″) corresponds to the viscous or liquid-like characteristics of the sample’s internal network [1]. In all formulations, G′ was consistently greater than G′′ across the entire frequency range, demonstrating a gel-like behaviour. Both G′ and G″ increased with angular frequency, suggesting that at higher oscillation speeds, molecular chains have less time to relax. This resulted in more transient entanglements behaving like cross-links, thereby enhancing the elasticity of the material [66]. Hybrid patties containing plant proteins exhibited distinct viscoelastic properties compared to the beef control. The higher G′ and G″ values observed in hybrid beef patties compared to the control have likely resulted from attractive interactions among fibre and starch components present in the plant proteins, contributing to a more solid-like behaviour in these formulations [67]. Notably, patties formulated with FBP showed the highest G′ and G″ values, which may be attributed to the high carbohydrate content of FBP. However, increasing the pre-hydration level of FBP led to reductions in both G′ and G″, indicating decreased viscoelasticity [68]. This decline is likely due to weakened protein–water interactions at higher hydration levels. These findings align with observed trends in WHC, which are known to influence rheological behaviour [1]. Furthermore, patties containing non-hydrated PP displayed higher viscoelastic moduli than those formulated with non-hydrated RP. This observation supports previous findings suggesting that pea protein enhances fat dispersion and emulsion stability in hybrid meat systems [25], thereby increasing G′ and G″ values [1]. Overall, the rheological differences observed were influenced by both types of plant protein and their pre-hydration level, highlighting the importance of formulation design in achieving desired texture properties in HPM products.

### 3.5. Cooking Loss

Cooking loss reflects the ability of patties to retain water during thermal processing, which causes protein denaturation and aggregation [17]. It is closely associated with key sensory attributes such as juiciness, flavour and tenderness [69]. As shown in Table 3, patty formulation had a significant effect on cooking loss (*p* < 0.05). All FBHP, PHP (0), and PHP (0.5) exhibited significantly lower cooking loss compared to the beef patty (30.2%) (*p* < 0.05). Although PHP (0.25) and all RHP also showed reduced cooking loss, these differences were not statistically significant (*p* > 0.05). The higher cooking loss observed in the beef control can be attributed to the thermal denaturation of myofibrillar proteins such as myosin and actin, along with the gelatinisation of collagen [70]. These structural changes cause the muscle fibres to shrink, leading to expulsion of water and fat from the protein matrix. In contrast, many plant proteins are already partially denatured during their extraction and processing [70]. As a result, the protein network in hybrid patties undergoes less collapse upon heating and instead forms a porous but stable matrix that may contribute to the retention of moisture and fat [70]. In addition, the presence of carbohydrates, particularly fibres and polysaccharides, in plant-based ingredients further contributes to forming a network that entraps fluids [19,20,21]. Additionally, higher WBC and OBC values of FBP and PP compared to RP support the lower cooking losses observed in FBHP and PHP. Furthermore, plant proteins have been reported to act as fat-encapsulating agents, mitigating oil dripping during cooking [71].

Therefore, the lower cooking loss observed in FBHP can be attributed to the high carbohydrate content (>60%) of FBP, which includes both starches and fibres. These components possess strong water-binding and fat-binding capacities, particularly during thermal processing, where starch gelatinisation and fibre swelling further enhance fluid retention. In contrast, the relatively higher cooking loss observed in RHP may be attributed to the greater surface hydrophobicity and lower solubility of rice proteins, which limit their integration into the myofibrillar protein matrix. As a result, these proteins may remain suspended in the aqueous phase rather than forming stable gels [64]. Additionally, increasing the pre-hydration level of each plant protein slightly increased cooking loss; however, the values remained consistently lower than those of the beef control, suggesting that even at higher hydration levels, hybrid formulations maintained superior water and fat retention.

### 3.6. Proximate Analysis and a_w_ of Raw and Cooked Patties

**Figure 4:**
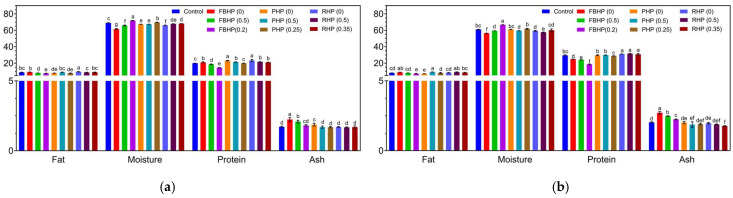
Proximate analysis (%) of (**a**) raw and (**b**) cooked beef/hybrid patties. ^a–f^ Means that do not share a common superscript are significantly different at *p* < 0.05. FBHP, PHP, and RHP represent faba bean, pea, and rice hybrid patties, respectively. Data are presented as mean ± standard deviation.

Proximate analysis and a_w_ of beef and hybrid patties formulated with FBP, PP, and RP at three pre-hydration levels are presented in Figure 4 and Table 3. Fat content ranged from 7.58% to 9.58% across all formulations. Although all patties were formulated to contain appropriately 10% fat, variations occurred due to differences in the fat content of plant-based protein ingredients and the use of 95% visual lean beef [72]. A decreasing trend in fat content was observed with increasing pre-hydration levels, likely due to the dilution effect from the added water. After cooking, FBHP (0) exhibited a higher fat content than the beef control, which may be attributed to its higher carbohydrate content. The carbohydrates likely formed a gel-like matrix during heating, effectively entrapping fat and reducing its loss during cooking. Moisture content was affected by the addition of hydrated plant protein slurries. Higher pre-hydration levels led to increased moisture in raw patties. However, control patties generally had significantly higher moisture content (*p* < 0.05) than hybrid formulations, except for FBHP (0.2) and PHP (0.25). This reduction in hybrid patties is likely due to the partial replacement of high-moisture beef with plant proteins, which had relatively low inherent moisture content (3.46–8.29 g/100 g). Similar trends were reported in hybrid chicken burgers formulated with pulse protein, where increased substitution reduced moisture content due to the strong water absorption capacity of pulses [20]. After cooking, moisture levels decreased across all samples, ranging from 56.44% to 66.68%. FBHP (0.2) retained the highest moisture content, reflecting the effect of both higher initial pre-hydration and water-holding properties of FBP. This is likely attributed to the high carbohydrate content in faba bean-based ingredients. These components can bind water physically (through swelling) or chemically (via hydrogen bonding), thereby helping to retain moisture during cooking. Protein content in raw patties ranged from 14.46% to 23.33%, with the lowest value observed in FBHP (0.2). This is attributed to the lower protein concentration in FBP, the dilution effect from higher pre-hydration levels, and the formulation design. Ash content in raw patties ranged from 1.67% to 2.24%, with FBHP showing significantly (*p* < 0.05) higher values, likely due to the higher ash content in FBP (5.54%) compared to RP (1.09%) and PP (3.64%). a_w_, a key indicator of microbial stability and food safety [73], was significantly (*p* < 0.05) higher in all hybrid patties compared to the control, except for FBHP (0). The lower a_w_ in FBHP might be due to the presence of low molecular weight carbohydrates in the FBP [1]. Increasing the pre-hydration level of plant proteins resulted in a rise in a_w_. This aligns with previous findings suggesting that plant-based meat analogues tend to exhibit higher a_w_ and weakly acidic pH values, which may increase the risk of microbial spoilage [73,74].

### 3.7. Water and Fat Retention and WHC

Moisture and fat retention values, which reflect a patty’s ability to retain water and fat during cooking, are summarised in Table 3. These factors are closely associated with cooking loss, texture, and sensory attributes of patties [71]. The addition of FBP and PP significantly (*p* < 0.05) improved moisture retention compared to beef patties (42.56%), while RP also showed an increase, although the difference was not statistically significant (*p* > 0.05). This improvement may be attributed to the high water-binding capacity of components in plant ingredients; for example, pea flour has been reported to be able to hold nearly five times its weight in water [75]. Similarly, patties formulated with FBP and PP showed significantly higher fat retention (*p* < 0.05) than the control patties (61.46%). In contrast, RHP demonstrated the lowest moisture and fat retention among all hybrid formulations. This may be due to the structural limitations of RP, which is less effective at forming a gel matrix during cooking, leading to higher cooking loss. WHC, defined as the ability of a sample to retain its inherent water during processing [51], was significantly higher (*p* < 0.05) in hybrid patties with plant proteins that were either non-hydrated or only slightly pre-hydrated. This suggests that lower pre-hydration levels allow plant proteins to bind water more effectively, likely due to better availability of functional binding sites. These results are consistent with the results of Hidayat, Wea, and Andriati [61], who reported that the addition of texturised vegetable protein (TVP) enhanced WHC in beef sausages. This is attributed to the higher content of water-soluble proteins and lower fat content in TVP compared to beef, allowing for more efficient water retention. Improved WHC reduces the presence of free water in the hybrid matrix, thereby contributing to lower cooking loss [51]. Additionally, variations in T_2_ relaxation times observed in the LF-NMR data support the differences in water distribution and mobility, offering a mechanistic explanation for the observed WHC differences across formulations.

### 3.8. Colour Analysis

**Figure 5:**
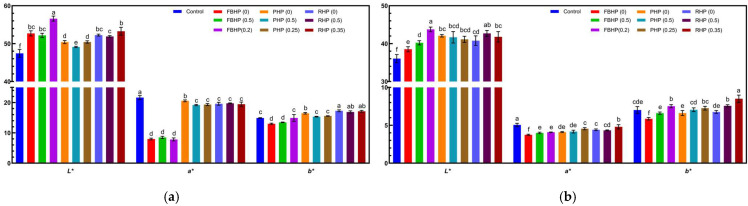
Colour analysis of (**a**) raw and (**b**) cooked beef/hybrid patties. ^a–f^ Means that do not share a common superscript are significantly different at *p* < 0.05. FBHP, PHP, and RHP represent faba bean, pea, and rice hybrid patties, respectively. L*, a*, and b* indicate lightness, redness/greenness, yellowness/blueness, respectively. Data are presented as mean ± standard deviation.

Colour is a critical quality attribute in meat products, as it significantly influences consumer perception and purchasing decisions. The colour parameters of both raw and cooked beef and hybrid patties are presented in Figure 1 and Figure 5. Incorporation of plant-based ingredients resulted in significant (*p* < 0.05) changes in instrumental colour parameters of patties. Raw hybrid patties exhibited significantly (*p* < 0.05) higher L* values and lower a* values compared to the beef control. These findings are consistent with previous studies, which reported that partial replacement of meat with plant-based ingredients increased L* due to the intrinsic colour of plant proteins [24]. In particular, hybrid patties formulated with higher pre-hydration levels demonstrated higher L* values, likely due to increased moisture content, which can enhance surface light reflection [24]. a* values were significantly reduced (*p* < 0.05) in all raw hybrid patties, with the lowest values observed in formulations containing FBP. This decrease aligns with prior studies [11,12,16,21,76,77], and can be attributed to the reduced myoglobin content resulting from meat substitution, as well as the presence of plant pigments, particularly the original dark green colour of plant proteins [16,76]. Furthermore, patties formulated with higher pre-hydration levels, such as FBHP and RHP, exhibited more pronounced reductions in a* values. This observation corresponds with findings by Argel and Ranalli [21], who noted that increased hydration in pea flour-based systems reduced a* values. Significant increases in b* values (*p* < 0.05) were observed in RHP and PHP (0), which may be attributed to the presence of yellowish pigments, such as phenolic compounds (anthocyanins and flavanols), in rice and pea protein [78,79]. These colour alterations highlight the impact of both the type and hydration level of plant proteins on raw patty appearance. The redness index (RI = a*/b*) values (Table 5) also differed significantly (*p* < 0.05) among formulations. PHP patties exhibited RI values closer to the beef control compared to FBHP and RHP, suggesting that PP retains more of the characteristic red appearance.

After cooking, L* values remained significantly higher in hybrid patties relative to the control (*p* < 0.05), continuing the trends from the raw state. The beef control showed the highest a* values after cooking, while patties containing FBP displayed the lowest. Among all cooked samples, RHP (0.35) exhibited the highest b* value after cooking, whereas FBHP (0) recorded the lowest. Notably, b* values increased consistently with pre-hydration level across all protein types. To further quantify surface colour changes after cooking, the browning index (BI) was calculated. BI values were significantly (*p* < 0.05) higher in the beef control and RHP (0.35), indicating greater development of brown colour. Browning in cooked patties is primarily attributed to non-enzymatic transformations, including Maillard reactions, caramelisation, oxidation of lipids, or structural modifications of proteins [73].

Although these colour differences were evident instrumentally, previous studies have shown that panellists did not perceive significant colour deviations between cooked hybrid and control products during sensory analysis [80,81]. However, the replication of traditional “meaty colour” in hybrid meat products remains an important factor for consumer acceptance, particularly among conventional meat consumers. Therefore, future formulation strategies should consider improving the raw appearance of HPM products to enhance their appeal and market competitiveness.

### 3.9. Texture Profile Analysis

**Figure 6:**
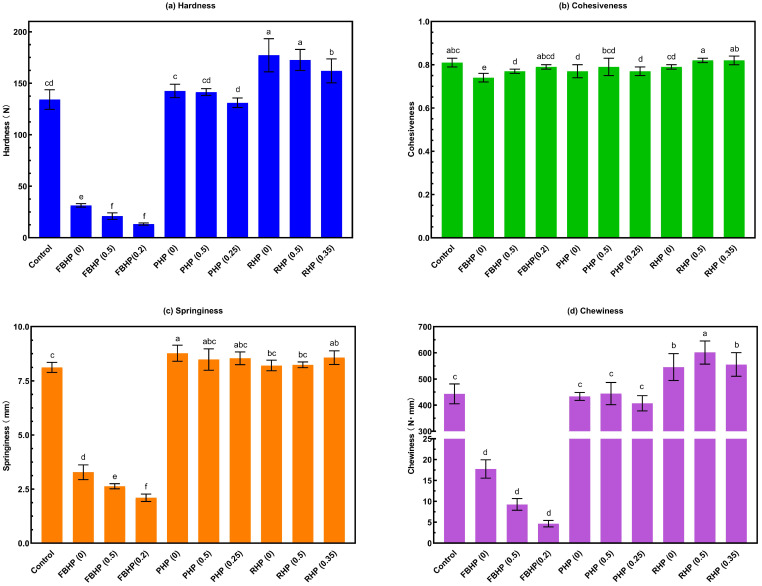
Texture profile analysis of beef/hybrid patties: (**a**) Hardness, (**b**) cohesiveness, (**c**) springiness, and (**d**) chewiness. ^a–f^ Means that do not share a common superscript are significantly different at *p* < 0.05. FBHP, PHP, and RHP represent faba bean, pea, and rice hybrid patties, respectively. Data are presented as mean ± standard deviation.

Texture is a critical quality parameter in meat products, influencing both eating quality and overall consumer acceptance. TPA results for hardness, cohesiveness, springiness, and chewiness are presented in Figure 6a–d.

Hardness varied significantly across samples (*p* < 0.05). PHP exhibited comparable hardness values compared to the control. While FBHP (0.2) demonstrated the lowest hardness (13.23 N), RHP (0) showed the highest (177.22 N). HPM products tend to exhibit lower hardness due to weaker intermolecular interactions among plant proteins [16] and structural disruptions in the protein matrix caused by non-meat proteins and carbohydrates [15]. For example, Nie, Xiong, and Jiang [25] reported that substituting 20–50% of meat with plant protein led to rougher textures and fragmented protein cross-linking. The high starch and fibre content of FBP (>60%) likely contributed to moisture and fat retention within the matrix [82], leading to reduced hardness. Lin and Barbut [64] similarly observed that faba bean proteins formed large but weakly connected aggregates in hybrid batters due to their high carbohydrate content. Conversely, the addition of rice protein increased the hardness of hybrid patties, likely due to its lower moisture content and poor emulsification properties, leading to phase separation during processing [31,55]. In general, the high hardness observed in cooked beef patties can be primarily attributed to the denaturation and thermal shrinkage of myofibrillar proteins such as myosin and actin [70]. Heat-induced protein unfolding and aggregation lead to contraction of the protein matrix and expulsion of fluids, resulting in increased protein–protein interactions and gel strength [70]. This structural tightening contributes to a firmer texture in beef patties. In contrast, the changes in textural attributes observed in hybrid patties were less pronounced. This may be because plant proteins are already partially denatured and aggregated during processing, limiting further thermal shrinkage or network tightening upon cooking [70]. Additionally, the carbohydrates present in the plant-based ingredients likely enhanced water retention during cooking. This not only reduced moisture loss but also contributed to a softer texture profile in the hybrid meat products. Additionally, higher pre-hydration levels were associated with reduced hardness across protein types. This may result from weaker network formation and increased moisture [13].

Cohesiveness of patties ranged from 0.74 to 0.82, with FBHP (0) showing the lowest value (0.74) and RHP (0.5) the highest (0.83). The observed increase in cohesiveness with higher pre-hydration levels indicates that pre-hydrated plant proteins enhance the binding capacity within the product matrix, contributing to improved structural integrity. Springiness is related to the height that the food recovers between the end of the first bite and the start of the second bite [83]. A higher springiness value means the food requires more chewing effort [84]. PHP (0) showed the highest springiness (8.77 mm), while FBHP (0.2) had the lowest (2.10 mm). The springiness of FBHP and PHP decreased as the pre-hydration level of plant proteins increased, while the springiness of RHP increased with higher pre-hydration levels. The reduction in springiness in FBHP and PHP could be attributed to increased fat and water retention [12], as FBP and PP effectively bind lipid and water droplets, potentially filling air gaps within the hybrid matrix and thereby reducing springiness. Springiness can be measured in several ways. In our study, springiness was recorded as the absolute distance (mm) that the sample recovered after the first compression, following the definition proposed by Bourne [53]. However, a more commonly used definition expresses springiness as a ratio, either of the time duration of force input during the second compression to that during the first compression, or of the detected height during the second compression divided by the original compression distance [83,85,86,87]. We acknowledge that differences in definitions and units can affect comparability across studies, and future work should consider adopting the ratio-based approach to align with the most widely accepted TPA methodology.

Chewiness is defined as the product of hardness, cohesiveness, and springiness. In this study, chewiness values of patties ranged from 4.66 to 601.49 N·mm and followed a trend similar to that of hardness. Higher chewiness values were typically observed in samples with both high hardness and springiness. For example, RHP (0.5) exhibited the highest chewiness, whereas FBHP (0.2) had the lowest. These differences are influenced by the type and hydration level of the incorporated plant proteins.

From the TPA, it is clear that the textural properties of hybrid patties became progressively softer, less chewy, and less cohesive in the following order: RHP > PHP > FBHP. FBHP exhibited lower hardness and chewiness values, while rice protein hybrid patties had the highest hardness, consistent with previous findings [19]. Additionally, increasing the pre-hydration level of plant proteins in hybrid patties reduced hardness. These textural differences may influence consumer satisfaction, as prior research has indicated that a moist texture enhances the acceptance of vegan and hybrid sausages [80]. Similarly, another survey involving 381 participants found that 30% of assessors preferred a “Soft” burger texture, while 21% selected “Firm” as their preferred attribute for an ideal burger [10]. Softness was identified as a desirable attribute in hybrid burgers due to its association with juiciness, which was rated as an ideal attribute by 82% of participants [10]. These findings underscore the importance of achieving a balanced texture profile in hybrid patties to optimise consumer satisfaction and market acceptance.

#### PCA

**Figure 7:**
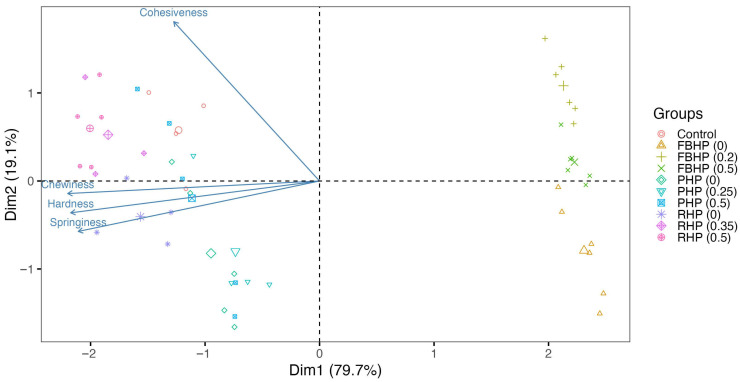
PCA plot of beef/hybrid patties. The x-axis (Dim1) and y-axis (Dim2) represent the first two principal components. Dim1 accounts for 79.7% of the total variance in the data, while Dim2 accounts for 19.1%. Each point on the plot represents a sample. The arrows indicate the direction and strength of each texture variable in the PCA space. The length of each arrow shows the importance of the variable in differentiating the samples along the principal components. Samples that are close to each other on the plot are similar in terms of the variables measured. The direction of the vectors shows the correlation between the variables and the principal components. Variables pointing in the same direction are positively correlated, while those pointing in opposite directions are negatively correlated.

PCA conducted on the textural properties of different formulations shows insights into the variability among formulations (Figure 7). The analysis is based on the first two principal components (Dim1 and Dim2), which together account for 98.8% of the total variance. The PCA plot shows clear clustering of specific formulations, indicating similarities in their textural properties. Formulations that cluster together show similar characteristics, while those that are well-separated along Dim1 or Dim2 show significant differences. For instance, control is distinctly separated from all other formulations, suggesting unique textural properties that are markedly different from the others. FBHP formulations, especially FBHP (0.2) and FBHP (0.5), are associated with higher cohesiveness and lower hardness, springiness, and chewiness. In contrast, the control group is characterised by a firmer texture profile with higher values in these attributes. PHP formulations, including PHP (0), PHP (0.25), and PHP (0.5), are closely clustered, indicating that varying pre-hydration levels of pea protein result in similar textural properties. This clustering suggests that the PHP formulations share a unique set of textural characteristics that differentiate them from the control and FBHP formulations. RHP formulations, such as RHP (0), RHP (0.35), and RHP (0.5), also display clustering, although with greater variability. The spread within the RHP cluster indicates slight variations in textural properties across RHP with different pre-hydration levels, but overall similarity in their texture profiles.

## 4. Conclusions

This study evaluated the technological properties of hybrid beef patties formulated with plant protein ingredients originating from three commercially relevant plant species at a 12.5% substitution level, with varying pre-hydration levels. The results showed that proteins and carbohydrates present in the incorporated plant-based ingredients, which possess water- and fat-holding capacities, contributed to the reduced cooking loss observed in the hybrid beef patties compared to the control. Although cooking loss increased with higher pre-hydration levels of plant-based ingredients. Among the proteins, FBP enhanced moisture retention and produced softer textures, likely due to its higher carbohydrate content. PP delivered texture properties most similar to the control beef patties, while RP led to firmer textures and lower moisture retention. In terms of colour, plant proteins raised the L* value and lowered the a* value, while the effect varied for the b* values among hybrid patties. The LF-NMR results showed that higher pre-hydration levels increased water mobility, indicated by raising T_2_ values. Rheology was affected by plant protein type and correlated with WHC.

Overall, the addition of various plant proteins significantly affects the physicochemical and functional properties of HPM products. PP showed the greatest potential for achieving meat-like texture in hybrid patties, while FBP with high carbohydrate content may appeal to consumers who prefer juicier and softer products. RP could be useful in formulations targeting firmer structure. Developing hybrid meat products with improved consumer acceptability requires a deeper understanding of the technological properties contributed by different plant proteins. Therefore, optimising the physical and functional properties of plant proteins could enhance the texture and overall quality of HPM products. Future studies should include detailed analyses of plant protein characteristics to better understand their roles in protein-meat matrix interactions and their impact on final product properties. Furthermore, strategically combining different plant protein sources could harness their complementary functional and nutritional properties, potentially enabling the development of hybrid formulations with improved texture, nutritional, and sensory qualities.

## Figures and Tables

**Table 1:** Experimental formulations for control and hybrid patties.

Formulation No.	Formulation Code	Formulation	Plant Ingredient	Pre-Hydration Rate of Plant Protein Ingredient in Water (*w*/*v*)
1	Control	Beef patty	NA	NA
2	FBHP (0)	Faba bean hybrid patty with dry FBP (no pre-hydration)	FBP	0
3	FBHP (0.5)	Faba bean hybrid patty with hydrated FBP	FBP	0.5
4	FBHP (0.2)	Faba bean hybrid patty with hydrated FBP	FBP	0.2
5	PHP (0)	Pea hybrid patty with dry PP (no pre-hydration)	PP	0
6	PHP (0.5)	Pea hybrid patty with hydrated PP	PP	0.5
7	PHP (0.25)	Pea hybrid patty with hydrated PP	PP	0.25
8	RHP (0)	Rice hybrid patty with dry RP (no pre-hydration)	RP	0
9	RHP (0.5)	Rice hybrid patty with hydrated RP	RP	0.5
10	RHP (0.35)	Rice hybrid patty with hydrated RP	RP	0.35

**Table 2:** Proximate compositions (g/100 g), a_w_, pH, WBC (g water/g dry sample), and OBC (g oil/g dry sample) analysis of plant proteins.

Properties	Faba Bean	Pea	Rice
Fat	0.94 ± 0.30 ^c^	4.61 ± 0.17 ^a^	1.81 ± 0.09 ^b^
Moisture	8.29 ± 0.08 ^a^	8.02 ± 0.04 ^b^	3.46 ± 0.07 ^c^
Protein	24.70 ± 0.14 ^c^	80.94 ± 0.13 ^a^	81.14 ± 0.03 ^a^
Ash	5.54 ± 0.30 ^a^	3.64 ± 0.05 ^b^	1.09 ± 0.03 c
Carbohydrate	60.53 ± 0.21 ^a^	2.79 ± 0.34 ^c^	12.51 ± 0.17 ^b^
a_w_	0.46 ± 0.00 ^b^	0.48 ± 0.00 ^a^	0.21 ± 0.01 ^c^
pH	4.90 ± 0.04 ^c^	7.52 ± 0.01 ^a^	6.74 ± 0.11 ^b^
WBC	3.69 ± 0.06 ^b^	4.70 ± 0.04 ^a^	1.73 ± 0.08 ^c^
OBC	2.34 ± 0.09 ^a^	0.99 ± 0.06 ^c^	1.24 ± 0.04 ^b^

Note: All data is the mean ± standard deviation of replicates. ^a–c^ Means in the same row that do not share a common superscript are significantly different (*p* < 0.05) from each other. a_w_: water activity, WBC: water-binding capacity, OBC: oil-binding capacity.

**Table 3:** Cooking loss, moisture retention, fat retention, WHC, pH, and a_w_ of beef/hybrid patties.

Beef/Hybrid Patties	Cooking Loss (%)	Moisture Retention (%)	Fat Retention (%)	WHC (%)	pH	a_w_
Control	30.20 ± 3.39 ^a^	42.56 ± 0.24 ^fg^	61.46 ± 2.19 ^e^	55.62 ± 4.33 ^de^	6.04 ± 0.16 ^abc^	0.975 ± 0.001 ^c^
FBHP (0)	13.28 ± 3.14 ^e^	48.95 ± 0.11 ^b^	81.94 ± 2.23 ^a^	78.02 ± 0.63 ^a^	5.55 ± 0.08 ^d^	0.975 ± 0.001 ^c^
FBHP (0.5)	18.95 ± 3.37 ^d^	48.13 ± 0.06 ^c^	83.61 ± 1.12 ^a^	74.61 ± 2.58 ^a^	5.38 ± 0.04 ^e^	0.980 ± 0.001 ^ab^
FBHP (0.2)	19.29 ± 0.01 ^d^	53.82 ± 0.14 ^a^	75.85 ± 5.31 ^b^	57.62 ± 1.56 ^de^	5.36 ± 0.04 ^e^	0.979 ± 0.001 ^ab^
PHP (0)	23.34 ± 1.68 ^cd^	46.80 ± 0.11 ^d^	68.36 ± 1.43 ^cd^	64.39 ± 3.77 ^b^	6.12 ± 0.04 ^ab^	0.979 ± 0.002 ^b^
PHP (0.5)	24.32 ± 0.32 ^bc^	43.47 ± 0.08 ^e^	76.58 ± 0.25 ^b^	58.75 ± 1.54 ^cd^	6.15 ± 0.06 ^a^	0.979 ± 0.002 ^b^
PHP (0.25)	27.51 ± 0.61 ^abc^	46.78 ± 0.38 ^d^	72.26 ± 5.78 ^bc^	59.57 ± 1.57 ^cd^	6.12 ± 0.05 ^abc^	0.982 ± 0.001 ^a^
RHP (0)	26.74 ± 0.59 ^abc^	42.99 ± 0.39 ^ef^	63.24 ± 2.52 ^de^	62.45 ± 1.76 ^bc^	6.02 ± 0.06 ^abc^	0.978 ± 0.000 ^b^
RHP (0.5)	28.54 ± 0.65 ^ab^	42.18 ± 0.19 ^g^	73.47 ± 2.53 ^bc^	53.89 ± 0.57 ^f^	5.94 ± 0.06 ^c^	0.979 ± 0.002 ^ab^
RHP (0.35)	26.99 ± 1.82 ^abc^	42.98 ± 1.09 ^ef^	68.66 ± 1.21 ^cd^	51.48 ± 0.73 ^f^	5.97 ± 0.04 ^bc^	0.979 ± 0.002 ^ab^

Note: All data is the mean ± standard deviation of replicates. ^a–g^ Means in the same column that do not share a common superscript are significantly different (*p* < 0.05) from each other. WHC: water-holding capacity. a_w_: water activity. FBHP, PHP, and RHP represent faba bean, pea, and rice hybrid patties, respectively.

**Table 4:** LF-NMR population distribution percentage of beef/hybrid patties.

Beef/Hybrid Patties	T_2b_ (ms)	T_21_ (ms)	T_22_ (ms)	P_2b_ (%)	P_21_ (%)	P_22_ (%)
Control	3.64 ± 0.20 ^c^	47.12 ± 0.00 ^a^	422.74 ± 87.17 ^b^	1.22 ± 0.24 ^ef^	97.08 ± 0.84 ^abc^	1.70 ± 0.71 ^bc^
FBHP (0)	2.90 ± 0.00 ^d^	24.04 ± 0.00 ^e^	187.02 ± 10.54 ^e^	6.19 ± 0.25 ^b^	90.41 ± 0.43 ^ef^	3.41 ± 0.21 ^a^
FBHP (0.5)	3.10 ± 0.17 ^d^	26.47 ± 0.00 ^d^	226.65 ± 12.77 ^de^	6.44 ± 0.26 ^b^	89.99 ± 0.57 ^f^	3.56 ± 0.76 ^a^
FBHP (0.2)	4.55 ± 0.25 ^a^	41.49 ± 2.26 ^b^	458.62 ± 25.03 ^b^	7.18 ± 0.24 ^a^	91.41 ± 0.32 ^e^	1.41 ± 0.31 ^c^
PHP (0)	3.75 ± 0.20 ^bc^	38.88 ± 0.00 ^c^	292.57 ± 0.00 ^cd^	1.68 ± 0.08 ^cd^	96.41 ± 0.32 ^cd^	1.90 ± 0.37 ^bc^
PHP (0.5)	3.00 ± 0.17 ^d^	42.80 ± 0.00 ^b^	431.04 ± 41.38 ^b^	1.33 ± 0.10 ^def^	97.60 ± 0.28 ^ab^	1.08 ± 0.19 ^c^
PHP (0.25)	3.10 ± 0.17 ^d^	47.12 ± 0.00 ^a^	654.34 ± 70.15 ^a^	1.08 ± 0.07 ^f^	98.17 ± 0.07 ^a^	0.74 ± 0.05 ^c^
RHP (0)	3.32 ± 0.50 ^cd^	42.80 ± 0.00 ^b^	428.81 ± 120.38 ^b^	1.56 ± 0.17 ^cde^	97.03 ± 0.72 ^bc^	1.42 ± 0.55 ^c^
RHP (0.5)	3.75 ± 0.20 ^bc^	42.80 ± 0.00 ^b^	312.24 ± 17.04 ^cd^	1.55 ± 0.25 ^cde^	95.75 ± 1.18 ^d^	2.70 ± 1.43 ^ab^
RHP (0.35)	4.13 ± 0.23 ^b^	47.12 ± 0.00 ^a^	368.79 ± 55.21 ^bc^	1.87 ± 0.25 ^c^	96.25 ± 0.61 ^cd^	1.88 ± 0.36 ^bc^

Note: All data is the mean ± standard deviation of replicates. ^a–f^ Means in the same column that do not share a common superscript are significantly different (*p* < 0.05) from each other. WHC: water-holding capacity. FBHP, PHP, and RHP represent faba bean, pea, and rice hybrid patties, respectively.

**Table 5:** Redness Index (RI) of raw patties and browning index (BI) of cooked patties.

Beef/Hybrid Patties	Redness Index (RI)	Browning Index (BI)
Control	1.45 ± 0.05 ^a^	31.55 ± 2.62 ^a^
FBHP (0)	0.61 ± 0.03 ^d^	23.28 ± 0.89 ^c^
FBHP (0.5)	0.63 ± 0.03 ^d^	24.85 ± 0.58 ^bc^
FBHP (0.2)	0.53 ± 0.01 ^e^	25.36 ± 0.95 ^bc^
PHP (0)	1.25 ± 0.01 ^b^	23.90 ± 1.01 ^c^
PHP (0.5)	1.25 ± 0.01 ^b^	25.55 ± 1.81 ^bc^
PHP (0.25)	1.24 ± 0.02 ^b^	27.20 ± 1.58 ^b^
RHP (0)	1.14 ± 0.01 ^c^	25.73 ± 1.44 ^bc^
RHP (0.5)	1.17 ± 0.03 ^c^	26.59 ± 0.56 ^b^
RHP (0.35)	1.14 ± 0.05 ^c^	30.85 ± 2.92 ^a^

Note: All data is the mean ± standard deviation of replicates. ^a–e^ Means in the same column that do not share a common superscript are significantly different (*p* < 0.05) from each other. FBHP, PHP, and RHP represent faba bean, pea, and rice hybrid patties, respectively.

## Data Availability

Data is contained within the article.
